# Trihexyphenidyl induced malignant hyperthermia in a patient with Parkinson's disease complicated with pneumonia: A case report

**DOI:** 10.1097/MD.0000000000020129

**Published:** 2020-05-15

**Authors:** Jun Zhao, Gang Xu, Congrui Feng, Yuluo Chen, Yanhong Kang, Feng Liu, Wei Ma

**Affiliations:** a Department of Geriatrics, National Clinical Key Specialty, Guangzhou First People's Hospital, School of Medicine, South China University of Technology; b Guangzhou First People's Hospital, Guangzhou Medical University; c Department of Respiration, The First Affiliated Hospital/School of Clinical Medicine of Guangdong Pharmaceutical University, Guangzhou, China.

**Keywords:** hyperthermia, refractory hyponatremia, syndrome of inappropriate secretion of antidiuretic hormone, trihexyphenidyl

## Abstract

**Introduction:**

Drug-induced fever is easy to overlook in respiratory departments. High fever is a rare side effect of trihexyphenidyl, which can be used clinically to treat Parkinson's disease. Syndrome of inappropriate antidiuretic hormone secretion (SIADH) is a group of clinical syndromes caused by various diseases, resulting in water retention and refractory hyponatremia. However, pneumonia combined with malignant hyperthermia and SIADH has rarely been reported. We describe an unusual case of malignant hyperthermia and refractory hyponatremia due to trihexyphenidyl adverse reaction.

**Patient concerns:**

Fifty-five-year-old male with pneumonia presented with malignant hyperthermia and refractory hyponatremia has a history of Parkinson's disease.

**Diagnosis:**

Early considerations related the described hyperthermia findings to the manifestations of pneumonia. However, the last findings were due to trihexyphenidyl adverse reaction.

**Interventions:**

Broad-spectrum antibiotics, oral and intravenous supplement of concentrated sodium chloride, drug, and physical cooling.

**Outcomes:**

The patient survived. During the 3-month follow up, the patient was no recurrence of fever or hyponatremia.

**Conclusion:**

High fever and SIADH can be a rare adverse reaction to trihexyphenidyl. Therefore, possible drug factors should be considered in the case. Consideration of other possible causes can improve early diagnosis and treatment of patients with fever of unknown origins.

## 1 Introduction

Fever of unknown origin is diagnosed following routine examination and treatment, in which the body temperature still has not restored to normal, and the diagnosis is not clear.^[1]^ Such fevers represent a difficult problem that is often encountered by clinicians, and determining the final diagnosis is difficult due to the wide variety of possible diseases leading to fever. When patients present with symptoms such as fever, cough, sputum production, lung wet rales, and hyponatremia, it is common to initially diagnose pneumonia, but the truth is often not so simple.

Trihexyphenidyl (benzhexol) is an anticholinergic drug that directly inhibits the parasympathetic nervous system and relieves muscle spasms.^[2]^ trihexyphenidyl is used clinically to treat Parkinson's disease, leading to improvements in stiffness, dyskinesia, tremor, and other symptoms.^[3,4]^ Along with this clinical application of trihexyphenidyl, adverse drug reactions caused by the drug are increasingly reported, including dry mouth, blurred vision, bradycardia, nausea, vomiting, urinary retention, constipation, and erythema.^[5,6]^ However, high fever is also a rare side effect of trihexyphenidyl.

The syndrome of inappropriate antidiuretic hormone secretion (SIADH) is a group of clinical syndromes caused by abnormal production of antidiuretic hormone (ADH), and is caused by various diseases. SIADH results in water retention, refractory hyponatremia, urinary sodium, and urinary osmotic pressure.^[7]^ SIADH is associated with the administration of certain drugs.^[8]^

Here, we report a unique case that was initially diagnosed as community-acquired pneumonia. The patient presented with high fever accompanied by SIADH, which we determined had been caused by trihexyphenidyl. We discuss the clinical implications of this case.

## 2 Case presentation

A 55-year-old man presented with high fever, cough, sputum production, and polypnea for 3 days. The patient had a body temperature of 39.2 degrees, with chills, a little fatigue, decreased appetite, and no other discomfort. His symptoms did not improve following oral administration of compound paracetamol tablets, with his temperature remaining above 39° with no sweat. The patient had a history of chronic sinusitis and Parkinson's disease diagnosed 6 years previously. For 3 months, he took 6 mg trihexyphenidyl three times per day (tid), the generic name is “Benzetholide hydrochloride,” and the drug specification is 2 mg/tablet, which is produced by Zhongnan Pharmaceutical Co., Ltd, Hunan province, China, 0.5 mg pramipexole hydrochloride tid, and 0.125 mg Madopar tid, which controlled his symptoms acceptably.

At admission, physical examination revealed a body temperature of 39.2°C, pulse rate of 76 beats per minute (bpm), and a breathing rate of 20 bpm. Moreover, breathing in the lower left lung sounded weaker with observation of some rales, while breathing in the rest of the lungs sounded clear without wet rales or pleural friction. Further assessments showed blood K^+^ levels of 3.20 mmol/L, Na^+^ of 118.2 mmol/L, CL^−^ of 86.3 mmol/L, and a 24-h urinary Na^+^ level of 554.7 mmol/day. Bloods revealed a white blood count of 11.58 × 10^9^/L, and a neutrophil level of 7.45 × 10^9^/L. Blood gas analysis showed hypoxemia and hyperventilation, with PaCO_2_ of 26.1 mm Hg, and PaO_2_ of 62.8 mm Hg.

Electrocardiogram and cardiac color ultrasound were not obviously abnormal. There was no evidence to indicate epidemic diseases, cancer, autoantibodies, or rheumatism. Paranasal sinusitis was apparent on skull computed tomography (CT). Meanwhile, pituitary magnetic resonance imaging showed no obvious lesions. A chest CT showed some inflammation of the left lower lobe and part of the fibrous formation. Full abdominal CT showed chronic cholecystitis and splenomegaly. There were no obvious abnormalities observed with positron emission tomography–CT.

At admission, the patient was diagnosed with community acquired pneumonia (CAP) and Parkinson's disease. For anti-infection treatment, he was administered piperacillin sodium and sulbactam sodium combined with azithromycin, and an oral and intravenous supplement of concentrated sodium chloride treatment for 1 week. Following this treatment, symptoms of cough, sputum production, and polypnea improved. However, the patient's body temperature rose to 41°C, and continued to present with refractory hyponatremia.

One day, on getting up the patient suddenly felt faint. He had a pale face, large dilatation of the pupils, and twitching of the upper limbs. His blood pressure dropped to 55/35 mm Hg, and doctors immediately implemented rescue procedures. After ∼1 min, the patient regained consciousness and could answer questions correctly. His blood pressure was 120/70 mm Hg, with a heart rate of 75 bpm, and his heart rhythm was regular with no murmur.

The fever did not abate after administration of antipyretic drugs, and therefore clinicians used an ice machine to cool the patient down. Surprisingly, the body temperature reduced to 37.5°C, but it rose again to 40°C after the ice machine was removed. Therefore, it was determined that the persistent high fever did not result from pneumonia but might be associated with a thermoregulatory central disorder.

The patient had been administered long-term, high dose oral trihexyphenidyl (6 mg tid). There are reports in the literature that excessive trihexyphenidyl can cause thermoregulatory central disorders, leading to high fever. Therefore, doctors stopped the trihexyphenidyl. After 3 days, the patient's body temperature gradually reduced to normal, and hyponatremia resolved. The patient was followed up for 3 months without fever or hyponatremia, and his general health was good.

## 3 Discussion

On the first day, patients are administered an oral dose of trihexyphenidyl of 1 to 2 mg, and this is subsequently increased by 1 to 2 mg every 2 to 5 days, up to a total dosage of 8 to 12 mg daily. The maximum dosage is 20 mg/day. trihexyphenidyl is associated with some central adverse reactions, including memory impairment, mental confusion, hallucinations, anxiety, sedation, and dyskinesia. Furthermore, peripheral adverse reactions are also common, including sweating disorders, dry mouth, blurred vision, and constipation.^[4,5,6]^ However, high fever is a rare side effect of trihexyphenidyl.

Because the patient had Parkinson's disease, he had taken a large dose of trihexyphenidyl (6 mg tid) for a long time. After the trihexyphenidyl was stopped, his temperature gradually returned to normal, and the patient remained without fever during 3 months of follow up. Therefore, we considered that the patient's high fever was caused by overdose of trihexyphenidyl.

The neuroleptic malignant syndrome (NMS) is a rare but potentially fatal disorder characterized by hyperthermia, muscle rigidity, and autonomic dysfunction and mental-status changes.^[9,10]^ The mechanism by which trihexyphenidyl induces hyperthermia may be related to interference of the thermoregulatory center, disturbed peripheral parasympathetic activity, inhibited secretion of sweat glands, and the loss of heat dissipation. The skin of the patient was always dry and sweat free, which contributed to the high fever. There are reports in the literature that trihexyphenidyl might cause thermoregulatory central disorders, leading to high fever. For example, Rajesh and colleagues reported that benzhexol caused high-grade fever, which is a clinical manifestation of NMS.^[11]^ These manifestations are due to dopaminergic transmission block in the hypothalamus and basal ganglia following dopamine antagonistic therapy.^[12,13]^ Moreover, Kwok and Chan^[14]^ reported a case of recurrent heat-related illness associated with the use of benzhexol, chlorpromazine, and zuclopenthixol decanoate. Moreover, several drugs can impair thermoregulation during exercise or under conditions of environmental heat stress. Indeed, anticholinergic drugs or drugs with anticholinergic effects can inhibit sweating and reduce heat elimination.

Interestingly, the patient reported here had refractory hyponatremia at the time of high fever, with serum sodium <135 mmol/L. Moreover, he had lower plasma osmolality with increased urine osmolality. The plasma osmolality was <280 mOsm/kg of water, while urine osmolality was greater than plasma osmolality (urinary sodium >20 mmol/day). No dehydration or edema was observed clinically. Moreover, heart, kidney, liver, adrenal gland, and thyroid gland function were normal, which accords with diagnostic standards for SIADH.^[15]^

SIADH is a group of clinical syndromes, such as water retention, diluent hyponatremia, urinary sodium, and increased urine osmolality, which are caused by the abnormal increase of ADH associated with various diseases.^[16]^ SIADH is common in diseases of the central nervous system (trauma, infection, hemorrhage, and cancer), malignant tumors (lung cancer, pancreatic cancer, lymphosarcoma, duodenal cancer, thymoma, etc.), pulmonary infections (tuberculosis and pneumonia), and may be associated with some drugs (chlorpropamide, carbamazepine, cyclophosphamide, tricyclic antidepressants, etc).^[17]^ The onset of SIADH is often unclear because early symptoms are not obvious and there is no specific signs. However, it can lead to persistent hyponatremia, which in severe cases causes nausea, vomiting, fatigue, brain cell edema, and death.^[18]^

In our case, the patient presented repeatedly with high fever, which was significantly associated with severe hyponatremia. Moreover, it was difficult to normalize serum sodium in the presence of high fever. After discontinuation of trihexyphenidyl, fever abated and serum sodium returned to normal, leading to improvements in symptoms. Therefore, we diagnosed SIADH resulting from treatment with trihexyphenidyl.

We hypothesize that high dose trihexyphenidyl can affect hypothalamic function, stimulating abnormal secretion of ADH or increased activity of ADH. In turn, this leads to water retention, increased urinary sodium excretion, and dilute hyponatremia, resulting in the occurrence of SIADH. As such, removing the influence of trihexyphenidyl caused SIADH symptoms to dissipate. During the 3-month follow up, there was no recurrence of fever or hyponatremia, and the prognosis was good.

In our review of international scientific literature, we did not find any reports of similar relevant cases. We suggest the cause of fever reported in this paper can be considered a rare adverse drug reaction. Drug-induced SIADH should be promptly treated, with treatment including:

1. withdrawal of the relevant drug(s);
2. water restriction and diuresis; and
3. appropriate sodium supplementation (0.5–1.0 mL/kg per hour 3% NaCl liquid).

The experience we get from this difficult case is that When a patient has fever accompanied by increased infection index, it does not mean that fever is caused by the infection, but it may also be caused by non-infectious factors.

## 4 Conclusion

In addition to considering infectious, neoplastic, and autoimmune diseases in the etiology of fever, drug factors should also be considered in patients presenting to respiratory departments. High-dose trihexyphenidyl may lead to the occurrence of high fever and SIADH. It is necessary to standardize the indications and dosage of trihexyphenidyl in clinics, with consideration of adverse drug reactions. Moreover, there should be early identification of SIADH and active treatment to avoid missed diagnosis and misdiagnosis.

## Author contributions

**Conceptualization:** Feng Liu, Wei Ma.

**Data curation:** Jun Zhao, Gang Xu, Congrui Feng.

**Formal analysis:** Jun Zhao, Gang Xu, Congrui Feng.

**Investigation:** Gang Xu, Yuluo Chen.

**Methodology:** Jun Zhao, Yanhong Kang.

**Resources:** Jun Zhao, Gang Xu, Yuluo Chen.

**Supervision:** Feng Liu, Wei Ma.

**Validation:** Feng Liu, Wei Ma.

**Writing – original draft:** Jun Zhao.

**Writing – review & editing:** Wei Ma.
